# T-wave Inversions in Cerebellar and Occipital Lobe Infarcts in the Setting of Deep Vein Thrombosis and Pulmonary Embolism Suggestive of Paradoxical Emboli: A Case Report

**DOI:** 10.7759/cureus.24230

**Published:** 2022-04-18

**Authors:** Mohsen S Alshamam, Nso Nso, Mahmoud Nassar, Zarwa Idrees, Victoria Ghernautan, Saifullah Khan, Yousef Abdalazeem, Most Munira

**Affiliations:** 1 Internal Medicine, Ichan School of Medicine at Mount Sinai / New York City (NYC) Health + Hospitals/Queens, New York, USA; 2 General Medicine, Saint James School of Medicine, St. Vincent, VCT; 3 East and North Hertfordshire NHS Trust, National Health Service (NHS), Hertfordshire, GBR; 4 Clinical Medicine and Cardiology, Ichan School of Medicine at Mount Sinai / New York City (NYC) Health + Hospitals/Queens, New York, USA

**Keywords:** case report, brain infarcts, acute cva, t-wave inversion, deep vein thrombosis (dvt), acute pulmonary embolism, cardio vascular disease

## Abstract

Cardiological causes account for the majority of acute electrocardiographic (ECG) changes. The reason for this fear is the irreversibility of myocardial necrosis. Generally, various changes can be observed in the ECG, including ST-T changes, QTc prolongation, arrhythmias, and T-wave inversions. Even though T-wave inversions can be seen in myocardial ischemia/infarction, they are rarely seen in acute cerebrovascular accidents (CVAs). We present the case of a 66-year-old woman who initially presented at our facility with dizziness in the context of orthostatic hypotension. An initial cardiac evaluation revealed no cardiac involvement. She was treated with intravenous fluids (IVF), which improved her symptoms. The patient's mental status was markedly altered approximately four days after admission. In this instance, she was found to have abnormal ECG findings (not previously observed on the ECG that was obtained on the day of admission), elevated troponin T levels, as well as elevated pro-B-type natriuretic peptide (pro-BNP). The patient was given aspirin and clopidogrel immediately and was placed on a heparin drip for a suspected non-ST elevation myocardial infarction (NSTEMI). A non-contrast computed tomography of the head revealed an acute cerebrovascular accident (CVA), following which the heparin drip was stopped. The patient was then transferred to another acute care facility capable of performing neurosurgical interventions. Additionally, a computed tomography angiography (CTA) of the chest and lower extremities venous duplex showed bilateral pulmonary emboli and deep venous thrombosis (DVT), respectively.

## Introduction

Some patients with acute cerebrovascular accidents (CVAs) may present with electrocardiographic (ECG) changes of cardiological concern. The commonly seen changes include ST-T changes, QTc prolongation, and arrhythmias [1,2]. Although T wave inversion is also seen, it is less common than the former three, and its prevalence is estimated to be less than three percent in acute cerebrovascular accident (CVA) patients [3]. T wave inversions are mostly linked to CVAs affecting the middle cerebral artery (MCA); however, rarely such changes are seen in infarcts affecting other territories/regions of the brain. Although not confirmed, the ECG changes are believed to be related to infarcts affecting the insular cortex supplied by the MCA [3], which is an area that possesses cardiovascular regulatory potentials via the autonomic system [4]. Clinical presentation of such patients can be deceiving at times, as some may present with no neurological complaints and/or cardiological symptoms. Additionally, laboratory and ECG/echocardiographic workup can be positive in these patients, supporting a primary myocardial process further obscuring the correct etiology behind these changes. Given that myocardial ischemia/infarction management is different from that of CVA in the acute setting, it is prudent to rule out CVA as a cause of T wave inversions to avoid unnecessary workup and invasive and/or harmful management.

## Case presentation

In this case report, we present a 66-year-old female patient who experienced a cerebrovascular accident (CVA) in 2009 and was left with residual left lower extremity weakness. She suffered from coronary artery disease (CAD) after multiple stentings in 2007. Her past medical history includes insulin-dependent type II diabetes mellitus, hypertension, obesity, and hyperlipidemia. She presented with dizziness. Her dizziness is described as a darkening of vision upon standing, followed by syncope and post syncope confusion for the last four weeks after receiving her influenza vaccine. She also has been having non-bilious vomiting for the last three days. The patient's exercise tolerance has decreased from seven to four blocks over the last month. When sleeping, she uses two pillows but denies paroxysmal nocturnal dyspnea (PND). She denied fever, chills, headaches, chest pain, shortness of breath, cough, palpitations, abdominal pain, diarrhea, new neurological sensory or motor deficits, dysuria, hematuria, urinary/bowel incontinence, or tongue biting. She denied any allergies or history of smoking or drug use. Her home medications were: aspirin 81 mg daily, clopidogrel 75 mg daily, atorvastatin 20 mg at bedtime, and insulin Levemir® 40 units at night. Her vital signs were within normal limits (WNL) except for a blood pressure of 159/56. The patient was moderately obese with a BMI of 39.10 kg/m^2^ (normal range:** **18.5-24.9). She had dry oral mucosa, decreased breath sounds, and left lower extremity weakness unchanged from baseline. Upon assessing orthostatic hypotension, the patient became symptomatic (dizziness, nausea, vomiting), necessitating early termination of the exam.

**Figure 1:**
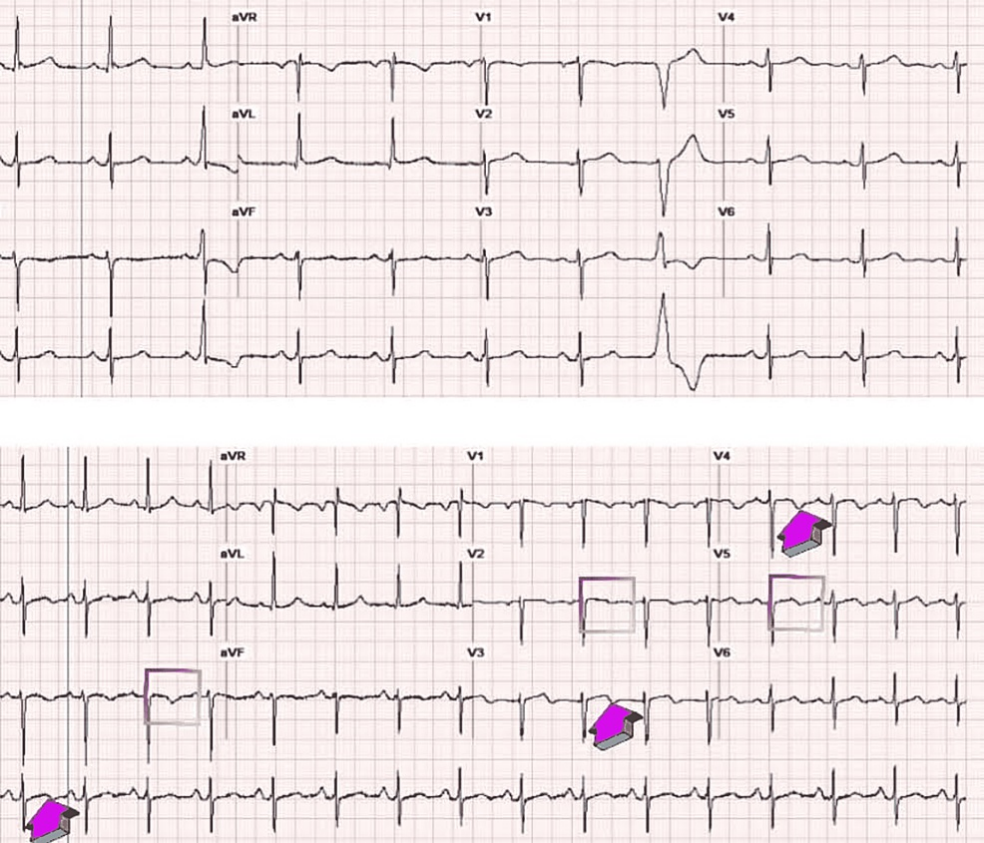
ECG/EKG Top image: sinus rhythm with PVCs but no acute ischemic changes. Bottom image: sinus rhythm with LAD and new T wave inversions/changes in leads II, III, V2-V5. Arrowheads and squares indicate T wave inversions/changes. ECG/EKG - electrocardiogram; PVCs - premature ventricular complexes; LAD - left axis deviation

**Figure 2:**
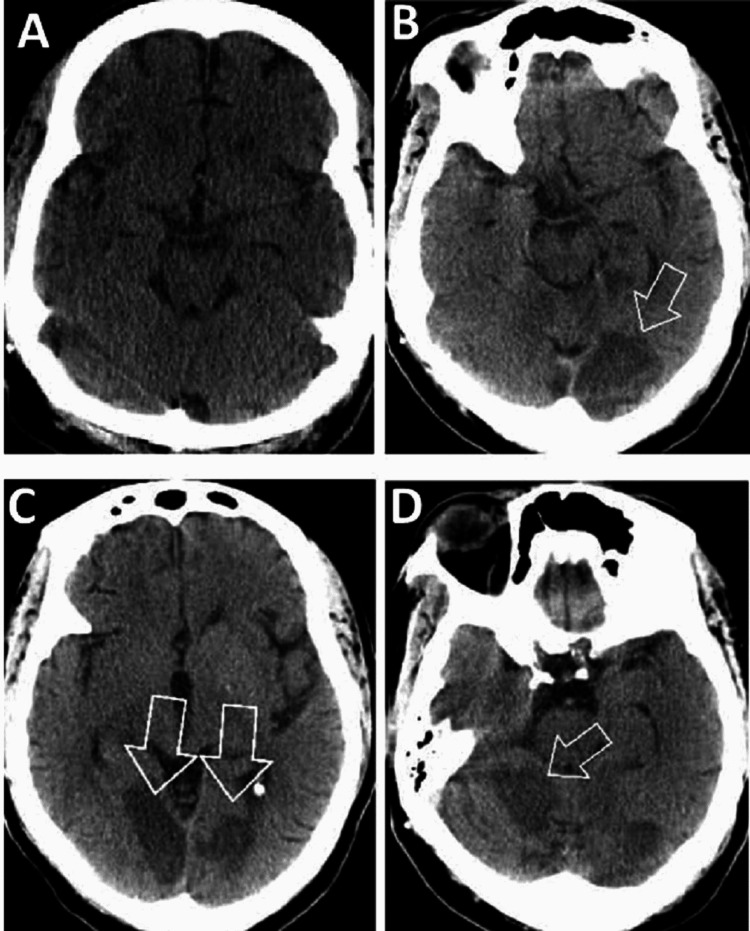
CT of the head without contrast at the time of admission and four days after admission A: CT of the head without contrast at the time of admission did not show any acute or subacute ischemic changes. B, C, and D: CT scans of the head without contrast four days after admission showed multifocal infarctions in the bilateral cerebellum, occipital lobe, and posterior left temporal lobe (arrowheads indicate infarctions).

**Figure 3:**
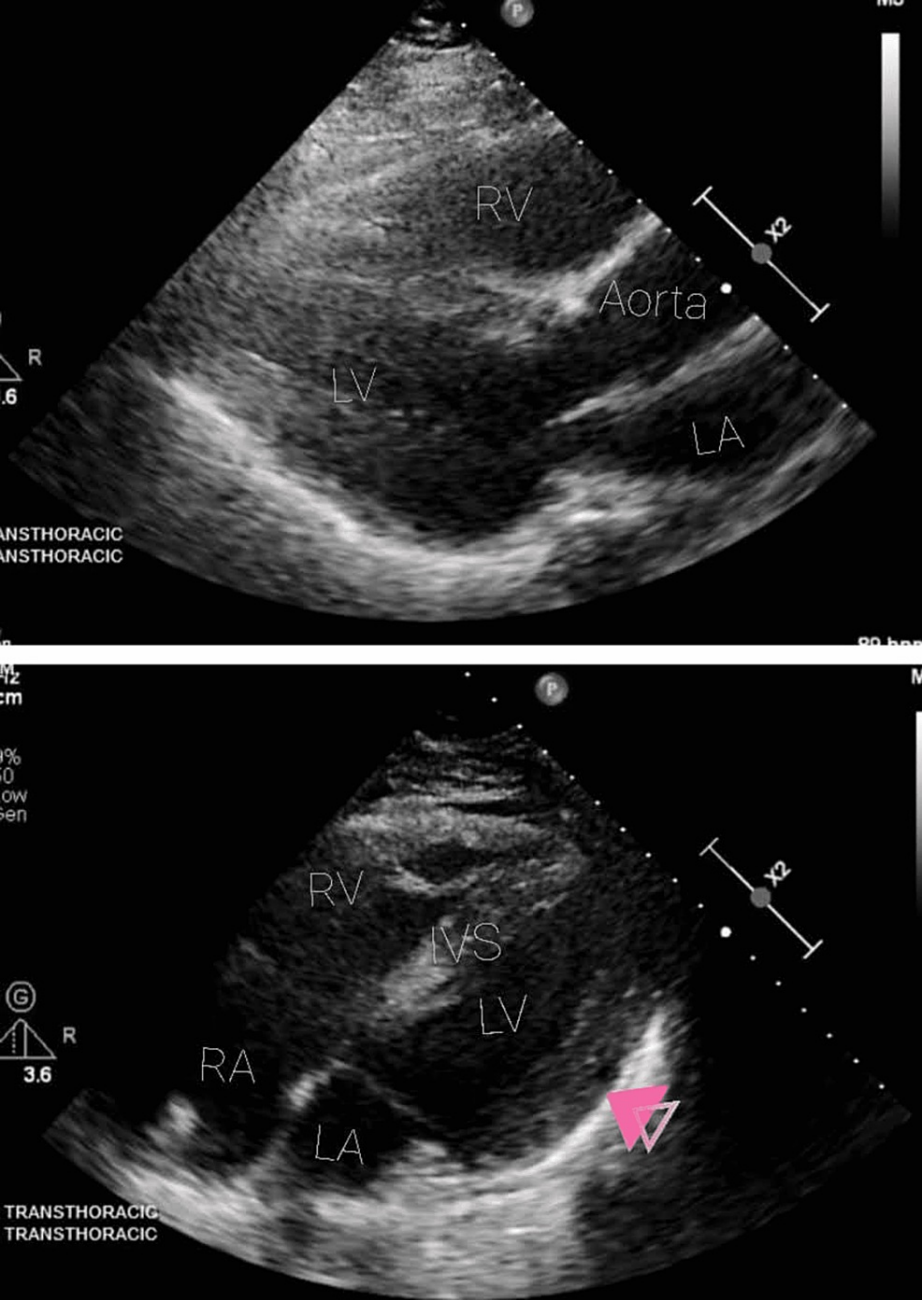
Transthoracic echocardiogram Top image: parasternal long-axis view of the heart. Bottom image: apical four-chamber view of the heart. Arrowhead indicates left ventricular hypertrophy (LVH). LA - left atrium; RA- right atrium; LV - left ventricle; RV - right ventricle; IVS - interventricular septum

The initial workup was essentially unremarkable, with an ECG showing normal sinus rhythm (NSR) at a rate of 62 beats per minute (BPM) and occasional ventricular premature complexes (PVCs) (Figure 1 top image). Troponin and pro-B-type natriuretic peptide (Pro-BNP) levels in the patient were within normal limits. Additional laboratory tests were performed as described in Table 1. Her first non-contrast computed tomography of the head showed possible acute on chronic right sphenoid sinusitis, but otherwise, it was negative for any acute or subacute intracranial pathology (Figure 2); electroencephalogram (EEG) was normal. A transthoracic echocardiogram (TTE) was obtained, which showed left ventricular hypertrophy, but otherwise a normal ejection fraction (EF) of 65-70% and normal wall motion (Figure 3). Cardiology was consulted, who believed the symptoms were autonomic and/or orthostatic and recommended up titrating metoprolol to 25 mg twice daily to control her PVCs, intravenous fluids (IVFs), and neurology consultation. Heparin was administered subcutaneously to prevent deep venous thrombosis (DVT).

**Table 1:** Laboratory findings at admission and four days after admission

Laboratory findings	Units	Value at admission	Value at four days after admission
Troponin	ng/mL	≤0.01	0.138
pro-B-type natriuretic peptide (pro-BNP)	pg/mL	88	1799
D-dimer	ng/mL DDU	244	8830
Hemoglobin	g/dL	12.3	12.3
Hematocrit	%	39.7	39.7
White blood cells	10(3)/mcL	9.6	4.8
Platelets	10(3)/mcL	199	156
Sodium (Na)	mmol/L	140	138
Potassium (K)	mmoL/L	4.3	4.6
Magnesium (Mg)	mg/dL	2.5	2.2
Calcium (Ca)	mg/dL	8.9	9.1
Glucose	mg/dL	291	181
Blood urea nitrogen	mg/dL	26	17
Creatinine	mg/dL	1.73	1.66
Bicarbonate	mmol/L	20	21
Lactate	mmol/L	2.7	-
Procalcitonin	ng/mL	0.19	0.12

The aforementioned management was instituted, after which the patient's symptoms improved; however, four days after her admission, the patient's mental status was found to be altered acutely. Her cardiac enzymes were positive this time (Table 1), and her ECG was NSR with poor R wave progression, left axis deviation (LAD), and T wave inversions/changes on the inferior and anterolateral leads (II, III, aVF, V2-V5) (Figure 1 bottom image).

She was loaded with aspirin and clopidogrel. Heparin drip was started for a possible non-ST elevation myocardial infarction (NSTEMI). A repeat non-contrast computed tomography of the head showed new bilateral multifocal cerebellar infarcts and new bilateral occipital lobe infarcts, extending into the posterior left temporal lobe (Figure 2), after which the heparin was held.

The TTE showed a normal EF and no abnormal wall motion, but the right ventricular stain was concerning for a pulmonary embolism (Figure 3). Cardiology concluded that her T wave changes were secondary to her acute CVA and not a primary cardiac process. Computed tomography angiography (CTA) chest showed non-occlusive bilateral pulmonary emboli with minimal enlargement of the pulmonary artery.

**Figure 4:**
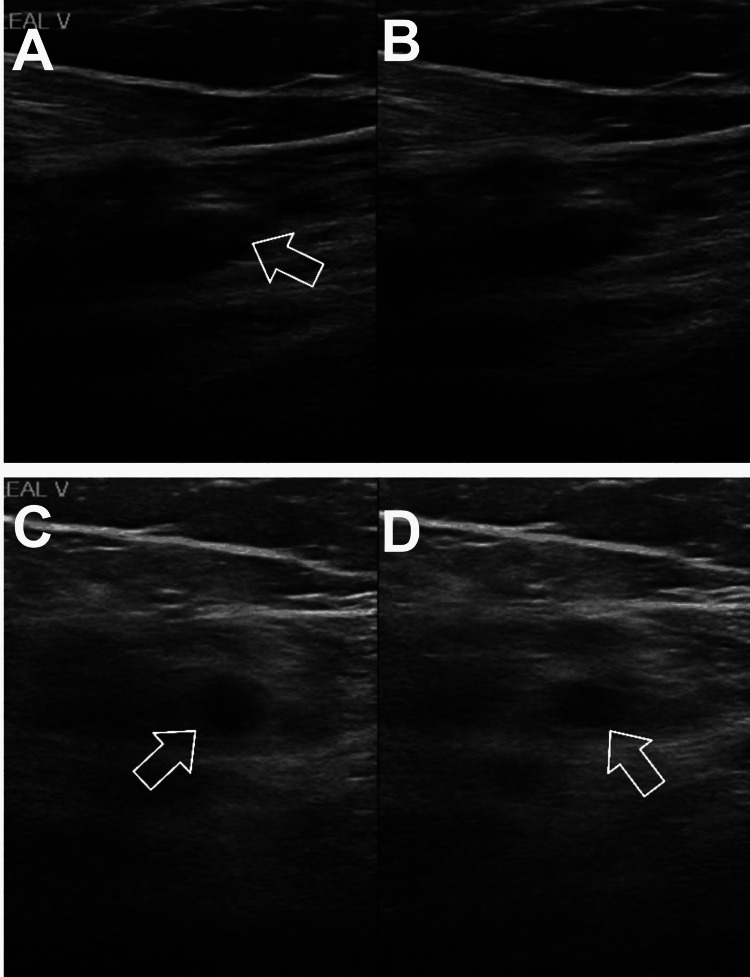
Lower extremity duplex Arrowheads indicate the left Soleal vein. B and D images before compression, A and C after compressions; on image A the arrowhead indicates an intraluminal thrombus.

Lower extremity (LE) venous duplex revealed acute left soleal occlusive deep vein thrombosis (DVT; Figure 4). Since the influenza vaccine was administered four weeks prior to the patient's hospital presentation, influenza is unlikely to be the cause of the patient's presentation. Sleep apnea was one of the differential diagnoses for altering mental status; consequently, outpatient sleep testing was scheduled. Nausea and vomiting could be signs of COVID-19 infection [5]. The patient had not received the COVID-19 vaccination. There were no signs of fever, chills, or respiratory illness, and oxygen was not required. The polymerase chain reaction (PCR) test for COVID-19 was negative, so it was ruled out. 

Systemic anticoagulation resulted in a hemorrhagic transformation of the stroke, further complicating her hospital course and necessitating a medical intensive care unit (MICU) admission. The patient was then transferred to an acute rehab facility approximately three weeks after her initial presentation.

## Discussion

Out of the many causes of ECG changes, acute CVA is a known yet uncommon and usually forgotten cause. ECG changes observed in CVA include ST-segment depression, QTc prolongation, atrial fibrillation, T wave inversions, Osborn J waves, and U waves [1,2]. Although ECG changes are observed in acute CVA, T wave inversions are not a common phenomenon. In a study of 800 patients admitted for hemorrhagic or ischemic strokes, 17 patients were found to have T wave inversions (2.1%). Sixty-five percent of such patients were found to have the primary insult to the MCA distribution, two had cerebellar strokes (12%), and two also were found to have PCA territorial infarcts (12%) [3]. Other cardiac arrhythmias, including PVCs, were observed as well as cardiac dysfunction (i.e., low EF, wall motion abnormality [WMA**]**), but the latter is very rare.

Several pathogenic processes have been proposed to explain the correlation between CVA and ECG findings, none of which has yet been established. One possible mechanism is that damage to the insular cortex, which is believed to be a region of cardiovascular autonomic regulation, is responsible for such ECG changes and possible physical myocardial damage. The MCA supplies this region; however, other territories not supplied by the MCA were described to have similar changes as well [3]. These patients often present with neurological deficits with concomitant ECG findings. However, non-neurological presentations were reported, which may mask the underlying diagnosis [6].

Another possible explanation of the constellation of findings (DVT, pulmonary embolism [PE], and CVA with ECG changes) is that there is a very high chance that the patient has had paradoxical emboli. Such diagnosis can be supported by ECG findings concerning right heart strain. Although there was no diagnostic workup performed to specifically identify any possible shunt that might have contributed to such phenomena, the presence of DVT/PE with arterial emboli without any corresponding left heart source makes the diagnosis of paradoxical emboli very likely [7].

One may argue that such changes could be related to PE, which our patient had. However, ECG changes favor CVA over PE, given that ECG changes in PE usually happen on the inferior and anteroseptal leads (II, III, aVF, V1-V4) and not the lateral leads (I, aVL, V5-V6). In our patient, we can see T wave changes in V5 and V6 as well. Another ECG hallmark supporting CVA is QTc prolongation, which is evidently seen on a follow-up ECG the next day. Additionally, T wave changes in CVA are said to be transient; the same is true regarding reversibility of ECG changes in PE, only in resolving right heart strain (i.e., thrombolysis/thrombectomy), which usually takes several days [8]. In our patient, a repeat ECG on the next day showed normalization of such changes, which would not have happened so quickly in the case of PE. Also, the sub-massive nature of the PE and the absence of clinical findings related to PE made our cardiology team associate such changes with CVA rather than PE.

Although it is possible that our patient had either thromboembolic or embolic phenomena, to our knowledge, no studies have been conducted to address which of the two causes such changes specifically. Furthermore, a weak argument can also be made that LVH, as well as a cardiac insult (NSTEMI), can cause similar changes; however, the absence and resolution of those changes a day before and a day after, respectively, with a TTE showing no wall motion abnormalities make this argument pointless.

In our patient, the ECG findings were of the rare type, T wave inversions, which were not caused by an MCA infarct, but by insults to the cerebellum bilaterally and the bilateral occipital lobes, which are rarely seen. Given the positive findings in our patient's CTA of the chest and LE venous duplex, it is possible that the ischemic intracranial changes were secondary to paradoxical emboli. Thus knowledge of T wave changes in various settings (i.e., cardiac ischemia/infarction, PE, hypertrophic cardiomyopathy [HCM], and CVA) is essential to correlate such changes with history and exam findings and execute related orders to formulate a correct diagnosis. The need for the right diagnosis is paramount to spare the patient from any unnecessary or harmful invasive procedures (i.e., cardiac catheterization) and potentially hazardous management (i.e., therapeutic anticoagulation in an acute CVA).

## Conclusions

Cardiologists are always concerned about ECG changes, particularly when elevated cardiac enzymes. Noncardiac causes can also present with similar changes, which can be misinterpreted as being cardiac in nature and delay the correct diagnosis. Therefore, clinical awareness and high suspicion of such changes in acute CVAs are essential, especially if the clinical presentation does not suggest a neurological condition. Early and accurate diagnosis protects patients from unnecessary and harmful invasive procedures and hazardous management.
